# Effects of integrative neuromuscular training on the gait biomechanics of children with overweight and obesity

**DOI:** 10.1111/sms.14163

**Published:** 2022-04-29

**Authors:** Pablo Molina‐Garcia, Alejandro Molina‐Molina, Annemie Smeets, Jairo H. Migueles, Francisco B. Ortega, Jos Vanrenterghem

**Affiliations:** ^1^ PROFITH “PROmoting FITness and Health Through PHYSICAL ACTIVITY” Research Group Department of Physical Education and Sports Faculty of Sport Sciences University of Granada Granada Spain; ^2^ Biohealth Research Institute Physical Medicine and Rehabilitation Service Virgen de las Nieves University Hospital Granada Spain; ^3^ Campus Universitario Universidad San Jorge Zaragoza Spain; ^4^ Research Group CTS‐545 ERGOLAB “Sport Physical Activity and Ergonomy to Life Quality” Department of Physical and Sports Education Faculty of Sports Science University of Granada Granada Spain; ^5^ Musculoskeletal Rehabilitation Research Group Faculty of Kinesiology and Rehabilitation Sciences KU Leuven Leuven Belgium; ^6^ Department of Biosciences and Nutrition Karolinska Institute Karolinska Sweden

**Keywords:** exercise therapy, flatfoot, gait analysis, musculoskeletal pain, pediatric obesity

## Abstract

**Objective:**

To analyze whether 13 weeks of integrative neuromuscular training can benefit spatiotemporal and kinematic parameters of gait in children with overweight/obesity.

**Methods:**

This is a non‐randomized controlled trial. Fifty children (10.77 ± 1.24 years, 31 girls) with overweight/obesity were allocated to an exercise group (EG) (*n* = 25) that carried out a 13‐week exercise program based on fundamental movement skills, strength activities and aerobic training, and a control group (CG) (*n* = 25) that followed their normal lifestyle. Spatiotemporal (i.e., cadence, stance and support times, step length, and stride width) and kinematic (i.e., hip, pelvis, knee, and ankle angles) parameters were evaluated under laboratory conditions through a 3D analysis. ANCOVA was used to test raw and z‐score differences between the EG and CG at post‐exercise, adjusting for pre‐exercise values.

**Results:**

The EG maintained their baseline stance and single‐limb support times while the CG increased them during walking (groups’ difference: 3.1 and 1.9 centiseconds). The EG maintained baseline maximum foot abduction angle during the stance phase whereas the CG showed an increase (groups’ difference: 3.9º). Additional analyses on kinematic profiles demonstrated that the EG walked with similar pelvic tilt and ankle abduction angles at post‐exercise, while the CG increased the pelvic anterior tilt in the whole stance phase (mean groups’ difference: 7.7º) and the ankle abduction angles in early‐ and mid‐stance phases (mean groups’ difference: 4.6º). No changes were observed in the rest of spatiotemporal and kinematic parameters.

**Conclusions:**

The integrative neuromuscular training stopped the progression of some biomechanical alterations during walking in children with overweight/obesity. These findings could contribute to preventing common movement‐derived musculoskeletal disorders in this population, as well as preserving an optimal mechanical efficiency during walking.

## 1 INTRODUCTION

Overweight/obesity (OW/OB) in childhood has risen alarmingly in the last decades in most countries around the world with severe consequences on the overall health of children.^1^ Among other consequences, OW/OB impairs daily locomotor activities of children, even very fundamental activities such as walking. A recent systematic review revealed biomechanical alterations during gait in this population, which could lead to the development of musculoskeletal disorders and energetic inefficiency during walking.^2^ In fact, these children and adolescents are more likely to experience pain and injuries,^3^, ^4^ while they require higher effort and absolute energy expenditure when walking than their normal‐weight peers.^5^, ^6^, ^7^

It is evident that the excess of body mass in these children plays a major role in the force parameters of gait (e.g., joint moments and contact forces), but some spatiotemporal (e.g., cadence and stride length) and kinematic (i.e., joint angles) parameters might also be affected.^2^ In fact, the abovementioned review revealed that children with OW/OB walk with longer stance time, greater step width and a more accentuated genu valgum position during the stance phase compared with their normal‐weight (NW) peers.^2^ Exercise interventions have been proposed as a promising treatment to combat these biomechanical alterations. However, to date we are only aware of three previous studies testing the effect of exercise on biomechanical gait parameters in children and adolescents with OW/OB.^8^, ^9^, ^10^

An 8‐week high‐intensity aerobic program had positive effects on gait speed and energetic efficiency in adolescents aged 13–16 years old with obesity.^10^ Two other exercise programs, one involving strength and neuromuscular training and the other one involving a yoga intervention (12 and 8 weeks of duration, respectively), found a reduction in the genu valgum during the stance phase of walking in children and adolescents with OW/OB.^8^, ^9^ Integrative neuromuscular training incorporates fundamental movements skills together with strength and conditioning tasks (e.g., resistance, balance, agility, or plyometric) for enhancing an integral motor skill development during childhood.^11^ This training approach is particularly interesting in children with OW/OB since have demonstrated a worsened motor development.^12^ We already observed that this integrative training leads to positive effects on plantar pressure, body posture, functional movement, and strength capacity in children with OW/OB.^13^, ^14^ However, it is unknown whether this exercise modality can be effective to diminish specific spatiotemporal and kinematics alterations derived from childhood obesity such as observed during walking. Thus, the main aim of the present study was to analyze the effect of a 13‐week integrative neuromuscular training on spatiotemporal and kinematic parameters of gait in children with OW/OB.

## 2 METHODS

### 2.1 Study design and participants

**FIGURE 1:**
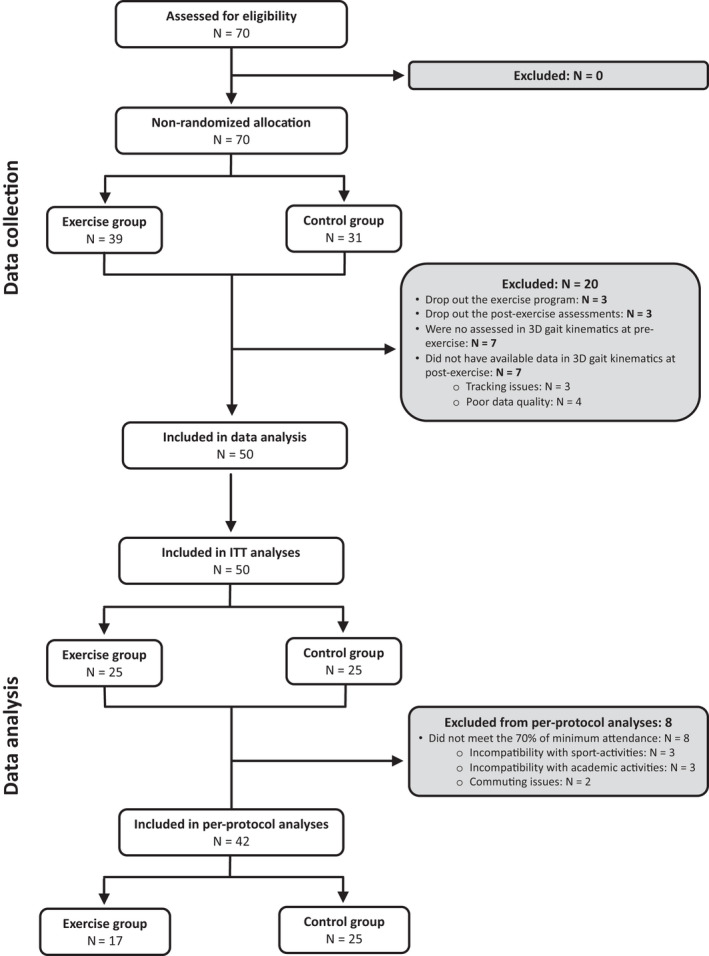
Flow diagram describing the data collection and data analysis processes

This study belongs to the MUBI (MUévete BIen in Spanish; Move Well in English) project, a non‐randomized controlled trial carried out in Granada (Spain) from February to July 2017. MUBI has been approved by the Ethics Committee on Human Research at the University of Granada (Reference: 279/CEIH/2017). MUBI is a nested study to a previous randomized control trial, the ActiveBrains project (Clinical trials. Gov identifier: NCT02295072).^15^ Those children who were randomly assigned to the CG in ActiveBrains were promised for ethical reasons to perform an exercise program the year after and then conformed the EG of MUBI. Thereafter, the CG of MUBI was recruited from primary schools in Granada (Spain) following the same inclusion/exclusion criteria as ActiveBrains. This CG also had the opportunity to participate in the exercise program after the study was finished. Fifty children between 8 and 12 years old (25.85 ± 3.58 kg/m^2^, 62% girls) from MUBI participated in this particular study after meeting inclusion/exclusion criteria previously published.^13^ Forty‐four children were included in the per‐protocol analysis, the primary analysis, and all 50 were included in the intention‐to‐treat analysis (Figure 1).

### 2.2 Three‐dimensional gait biomechanics

Three‐dimensional gait biomechanics were evaluated using a motion capture system composed by eight high‐resolution cameras (model mvBluecougar‐XD104C, Matrix Vision GmbH, Germany) operating at 100 Hz with a resolution of 2048 × 1088 pixels, the Simi Motion software v.9.2.2. (Simi Reality Motion Systems GmbH, Germany) and a twenty‐one marker model according to the International Society of Biomechanics (ISB) standard.^16^ This model demonstrates clinically acceptable intertrial repeatability in all reported joint angles, which allows the detection of changes after the intervention period.^17^ Before gait recording, a static trial in anatomic position was used to define anatomic coordinate systems for the foot, shank, thigh, and pelvis.^16^ Children walked barefoot during 15 s on a treadmill (Woodway Pro XL, Waukesha, WI, USA) at a self‐selected speed, which was determined in a prior familiarization trial. The same speed was used at post‐exercise to not influence kinematics since it affects to a greater extent in children with immature gait patterns.^18^, ^19^ Upright static trials and a minimum of seven valid gait cycles (i.e., from heel strike to heel strike of the same foot) were exported for subsequent analysis in Visual 3D software 4.96.11 (C‐Motion, Inc., Germantown, MD, USA). Five spatiotemporal parameters (i.e., cadence, stance and support times, step length, and stride width) and pelvis, hip, knee, and ankle kinematics in all three planes (i.e., sagittal, frontal, and transversal) were calculated. Data processing and calculations in Visual 3D are described in Appendix S1. Two experts in biomechanics with more than one year of experience conducted the assessment, and they were blinded to group (i.e., they did not know whether participants belong to the control or intervention group).

### 2.3 Musculoskeletal pain

The Pediatric Pain Questionnaire™ was used to identify self‐reported musculoskeletal pain.^20^ Children were instructed to highlight the areas where they usually feel pain on a body map. Four different colors were used to indicate the intensity of this pain (i.e., low, mild, moderate, and severe). Before the children filled in the questionnaire, an instructed evaluator explained the type of pain the children should report, and immediately after, each questionnaire was reviewed to discard non‐related musculoskeletal pain (i.e., head or stomach pain). Based on this questionnaire, children were categorized as follows: (1) presence of any pain intensity (“yes” or “no”) in knees, low back, and lower limbs (i.e., feet, knees, hips, or lumbar spine); and (2) presence of moderate‐to‐severe pain (“yes” or “no”) in the abovementioned regions.

### 2.4 Potential confounders

Body height and weight were measured to the nearest 0.1 kg and 0.1 cm, respectively (SECA Instruments, Germany), and body mass index (BMI, kg/m²) was calculated. The maturational stage of the children was determined by calculating the peak height velocity with Moore's equations, which use participants’ age and standing and sitting height.^21^

### 2.5 Exercise program

The exercise program was undertaken at the Sport and Health University Research Institute (iMUDS) between the March 1, 2017, and the May 29, 2017. Group sessions were offered from Monday to Friday, and participants were asked to attend a minimum of three sessions per week. The concept of integrative neuromuscular training is based on the simultaneous development of motor competence (e.g., fundamental movement skills), strength and conditioning (e.g., resistance training and core‐focused strength) and aerobic fitness in the same exercise program.^11^ Sessions lasted 90 min and were divided into two different parts: 30 min of movement quality work and 60 min of multi‐games. During the movement quality part, children acquired awareness in their movements (e.g., anterior and posterior pelvic tilt) and body posture (e.g., optimal spine position), they trained joint mobility (e.g., hip flexion mobility) and stability (e.g., core stability) to gain muscular strength over a functional range of motions (e.g., bilateral lower‐limb push strength), and they learned fundamental movement patterns (e.g., squat pattern). The multi‐games part of the exercise program aimed to reach a moderate‐to‐vigorous intensity of aerobic exercise, to teach children a wide range of fundamental movement skills (e.g., sprinting, hopping, or throwing), and to make physical exercise an enjoyable activity. Further information about the exercise program can be found elsewhere (http://profith.ugr.es/pages/investigacion/proyectos/rationaleexerciseprogram).

### 2.6 Statistical analysis

Baseline differences between the EG and the CG in all included outcomes were investigated by performing t‐tests for continuous outcomes and chi‐squared tests for categorical outcomes. Following CONSORT guidelines, we present both the per‐protocol and intention‐to‐treat analyses to interpret results from an overall perspective.^22^ The per‐protocol approach is presented as the main analysis in order to show the efficacy in those children who strictly adhered to the exercise program. Inclusion criteria in the per‐protocol analysis are as follows: (1) to complete the pre‐ and post‐intervention assessments, and (2) to attend to at least 70% of the recommended 3 sessions/week (i.e., exercise group) according with previous trials.^23^ Firstly, outcomes were checked for normal distributions through histograms. Secondly, pre‐exercise z‐scores were calculated, and post‐exercise z‐scores were based on this pre‐exercise score according to the following formula used in previous trials^24^: (participant's raw score at post‐exercise—sample's mean raw score at pre‐exercise)/sample's standard deviation at pre‐exercise. Thirdly, a one‐way analysis of covariance (ANCOVA) was used to examine differences in gait biomechanical outcomes between the EG and the CG at post‐exercise, and including pre‐exercise values as a covariate. Results from the ANCOVA were presented in raw and *z*‐scores. Raw data allow clinical interpretations while z‐score is an standardized measure interpreted as how many standard deviations (SDs) has changed the EG compared to the CG. This last is useful in determining the effect size, which is interpreted as follow: 0.2–0.5 SDs = small effect size; 0.5–0.8 SDs = medium effect size; and ≥0.8 = large effect size.^25^ Similarly, within‐group and between‐group pre‐minus post‐exercise differences were reported in raw and *z*‐scores to better understand how each group has changed with respect to itself between the pre‐ and post‐exercise assessment. Additional confounders such as gender, age, maturational status, anthropometric measures, and gait speed were discarded after verifying that these overall did not influence the ANCOVA models (all *p* > 0.05). However, sensitivity analyses were conducted with these confounders when significant results were found. Intention‐to‐treat analyses are presented in the Appendix S1 and followed the same process as explained above for the per‐protocol analysis.

Additionally, SPM1D package available for Matlab (v.0.4, http://www.spm1d.org) was used to investigate the effects of exercise on the entire gait kinematic curves during one stride cycle.^26^ SPM1D is a statistical tool using the random field theory and allows one to conduct conventional statistical tests on one‐dimensional data (e.g., kinematic curves). Firstly, a two‐way mixed ANOVA was performed to test the interaction effect between groups (EG vs CG) and assessment time (pre‐ and post‐exercise). Secondly, a post hoc analysis was performed for those kinematic parameters demonstrating an interaction effect, which consisted of paired SPM *t*‐tests comparing pre‐ and post‐exercise gait kinematics in each group (EG and CG). Considering the exploratory nature of this kinematic analysis, no corrections for multiple testing were performed to avoid overly conservative interpretations.

Lastly, chi‐square’ and McNemar's tests were used to examine pre‐ and post‐intervention differences in musculoskeletal pain within and between groups, respectively. Analyses were performed using the SPSS (version 24.0, IBM Corporation) and Matlab (version 9.5.0.1033004 [R2018], Mathworks, Inc.) software. The level of significance was set at *p* < 0.05.

## 3 RESULTS

Baseline characteristics of the participants included in the per‐protocol analysis (*N* = 42) are shown in Table 1. On average, children attended 71.6% of the recommended exercise sessions (i.e., 3 sessions per week). Fifty‐six percent of children presented an adherence above 70% and forty‐four percent above 95%, while thirty‐two percent showed an adherence between 18% and 50%. Between‐group differences (EG minus CG at post‐test adjusting by baseline) after the exercise program are shown in Table 2. In the spatiotemporal parameters, the EG showed a smaller increase in stance and single‐limb support times compared to the CG (raw values: −3.1 and −1.9 centiseconds; medium effect size: −0.55 and −0.73 SDs; *p* = 0.036 and 0.014, respectively). The remaining spatiotemporal parameters were not significantly different between groups (all *p* > 0.05). Regarding the gait kinematics, a significant between‐group difference in the maximal ankle abduction angle at weight acceptance was found (raw value: −3.9º; small effect size: −0.42 SDs; *p* = 0.012). More specifically, this angle did not change in the EG over time while it increased in the CG. No other between‐group differences were found for the other kinematic outcomes (all *p* > 0.05).

**TABLE 1:** Pre‐exercise characteristics of the per‐protocol sample and divided by intervention and control group

	All (*N* = 42)	Intervention (*N* = 17)	Control (*N* = 25)	*P*
	Mean ± SD	Mean ± SD	Mean ± SD	
Age (years)	10.9 ± 1.3	11.4 ± 1.1	10.5 ± 1.2	**0.021**
Weight (kg)	57.7 ± 12.4	64.8 ± 7.6	53.0 ± 12.9	**0.002**
Height (cm)	148.7 ± 8.6	152.5 ± 6.0	146.1 ± 9.3	**0.017**
Body mass index (kg/m^2^)	25.8 ± 3.6	27.8 ± 2.8	24.5 ± 3.4	**0.002**
Gender *N* (%)				
Girls	26 (62)	6 (35)	20 (80)	**0.003**
Boys	16 (38)	11 (65)	5 (20)	
Spatiotemporal parameters				
Cadence (steps/min)	122.5 ± 12.0	119.9 ± 7.9	124.3 ± 14.0	0.254
Stance time (cs)	66.7 ± 56.2	67.3 ± 5.1	66.2 ± 6.0	0.537
Single‐limb support time (cs)	32.9 ± 2.6	33.4 ± 2.0	32.6 ± 2.9	0.357
Double support time (cs)	33.7 ± 3.9	33.9 ± 3.8	33.6 ± 4.0	0.772
Step length (cm)	51.4 ± 8.4	56.1 ± 4.5	48.2 ± 9.0	**0.002**
Stride width (cm)	13.9 ± 3.2	13.6 ± 2.9	14.2 ± 3.5	0.555
Kinematics: stance phase (º)				
Pelvis ROM sagittal	4.5 ± 1.1	4.5 ± 1.1	4.5 ± 1.0	0.946
Knee ROM frontal	5.9 ± 3.6	6.5 ± 4.9	5.6 ± 2.3	0.438
Ankle max. plantarflexion	60.2 ± 9.7	62.5 ± 10.3	58.6 ± 9.1	0.206
Kinematics: weight acceptance (º)				
Pelvis max. elevation	3.6 ± 2.6	4.0 ± 2.9	3.4 ± 2.5	0.539
Hip ROM frontal	3.7 ± 2.1	4.2 ± 2.5	3.3 ± 1.8	0.192
Knee ROM sagittal	14.4 ± 5.5	16.1 ± 5.5	13.2 ± 5.3	0.091
Ankle max. abduction	13.8 ± 9.4	15.8 ± 10.4	12.4 ± 8.6	0.254

Note

Values are presented as mean ± SD or percentages. For continuous variables, *p* value was obtained by an independent samples *t*‐test, whereas for categorical variables, *p* value was obtained by chi‐square test. Significant differences (*p* < 0.05) are highlighted in bold; *N*, sample size.

Abbreviation: SD, standard deviation.

**TABLE 2:** Per‐protocol intervention effects on gait biomechanics

Adjusted post‐exercise mean (95% CI)				
Total sample = 42	Exercise group (*N* = 17)	Control group (*N* = 25)	Groups’ difference (EG–CG)	*p*
**Spatiotemporal parameters**				
Cadence (steps/min)				
Raw score	119.8 (115.6 to 124.1)	115.0 (111.5 to 118.5)	4.8 (−0.7 to 10.3)	0.088
*z*Score	−0.2 (−0.6 to 0.1)	−0.6 (−0.9 to −0.3)	0.4 (−0.1 to 0.9)	
Stance time (cs)				
Raw score	68.1 (65.8 to 70.3)	71.2 (69.4 to 73.0)	−3.1 (−6.0 to −2.1)	**0.036**
*z*Score	0.2 (−0.1 to 0.6)	0.8 (0.5 to 1.1)	−0.5 (−1.1 to −0.0)	
Single support time (cs)				
Raw score	33.8 (32.7 to 34.9)	35.6 (34.7 to 36.6)	−1.9 (−3.3 to −0.4)	**0.014**
*z*Score	0.3 (−0.1 to 0.8)	1.1 (0.7 to 1.4)	−0.7 (−1.3 to −0.2)	
Double support time (cs)				
Raw score	34.4 (33.0 to 35.7)	35.5 (34.4 to 36.6)	−1.1 (−2.9 to 0.6)	0.191
*z*Score	0.2 (−0.2 to 0.5)	0.5 (0.2 to 0.7)	−0.3 (−0.7 to 0.1)	
Step length (cm)				
Raw score	52.8 (50.4 to 55.2)	54.2 (52.2 to 56.1)	−1.3 (−4.6 to −1.9)	0.415
*z*Score	0.2 (−0.1 to 0.5)	0.3 (0.1 to 0.6)	−0.2 (−0.5 to 0.2)	
Stride width (cm)				
Raw score	14.1 (13.0 to 15.2)	13.4 (12.5 to 14.3)	0.7 (−0.7 to 2.1)	0.337
*z*Score	0.0 (−0.3 to 0.4)	−0.2 (−0.5 to 0.1)	0.2 (−0.2 to 0.7)	
**Kinematics (degrees): stance phase**				
Pelvis ROM sagittal				
Raw score	4.3 (3.7 to 5.0)	3.9 (3.4 to 4.4)	0.4 (−0.4 to 1.2)	0.308
*z*Score	−0.2 (−0.8 to 0.4)	−0.6 (−1.1. to −0.1)	0.4 (−0.4 to 1.2)	
Knee ROM frontal				
Raw score	8.4 (7.1 to 9.8)	7.6 (6.5 to 8.8)	0.8 (−1.0 to 2.6)	0.361
*z*Score	0.7 (0.3 to 1.1)	0.5 (0.2 to 0.8)	0.2 (−0.3 to 0.7)	
Ankle max. plantarflexion				
Raw score	56.7 (54.1 to 59.3)	57.2 (55.1 to 59.3)	−0.5 (−3.9 to 2.9)	0.756
*z*Score	0.4 (0.1 to 0.6)	0.31 (0.1 to 0.5)	0.0 (−0.3 to 0.4)	
**Kinematics (degrees): weight acceptance**				
Pelvis max. elevation				
Raw score	2.0 (1.0 to 2.9)	1.8 (1.0 to 2.6)	0.1 (−1.1 to 1.4)	0.826
*z*Score	0.6 (0.3 to 1.0)	0.7 (0.4 to 1.0)	0.0 (−0.4 to 0.5)	
Hip ROM frontal plane				
Raw score	3.7 (2.8 to 4.5)	3.3 (2.6 to 4.0)	0.3 (−0.7 to 1.4)	0.521
*z*Score	−0.0 (−0.4 to 0.4)	−0.2 (−0.5 to 0.1)	0.2 (−0.3 to 0.7)	
Knee ROM sagittal				
Raw score	13.7 (12.0 to 15.5)	14.7 (13.3 to 16.2)	−1.0 (−3.3 to 1.3)	0.371
*z*Score	−0.1 (−0.4 to 0.2)	0.1 (−0.2 to 0.3)	−0.2 (−0.6 to 0.2)	
Ankle max. abduction				
Raw score	14.4 (12.1 to 16.7)	18.2 (16.4 to 20.1)	−3.9 (−6.9 to 0.9)	**0.012**
*z*Score	0.1 (−0.2 to 0.3)	0.5 (0.3 to 0.7)	−0.4 (−0.7 to −0.1)	

Note

A one‐way analysis of covariance (ANCOVA) was used to test raw and *z*‐score differences between the EG and CG at post‐exercise, adjusting for pre‐exercise values. *Z*‐score values in the “group difference” column indicates how many standard deviations has changed the EG compared to the CG, and can be interpreted as an effect size indicator: 0.2–0.5 SDs, small effect size; 0.5–0.8 SDs, medium effect size; and ≥0.8 = large effect size (e.g., 0.51 *z*‐score means that the EG has changed +0.51 standard deviations compared to the CG, which is a medium effect size). *Z*‐score values in both “exercise group” and “control group” columns indicates how many standard deviations has changed each group with respect to itself between the pre‐ and post‐exercise assessment (e.g., 0.51 *z*‐score in the “exercise group” column means that the EG is 0.51 standard deviations higher at post‐exercise than at pre‐exercise). Adjusted means and confidence intervals of the mean are represented. Significant differences (*p* < 0.05) are highlighted in bold. *n* = sample size.

Abbreviations: CI, confidence interval; EG, exercise group; CG, control group; cs, centiseconds.

**FIGURE 2:**
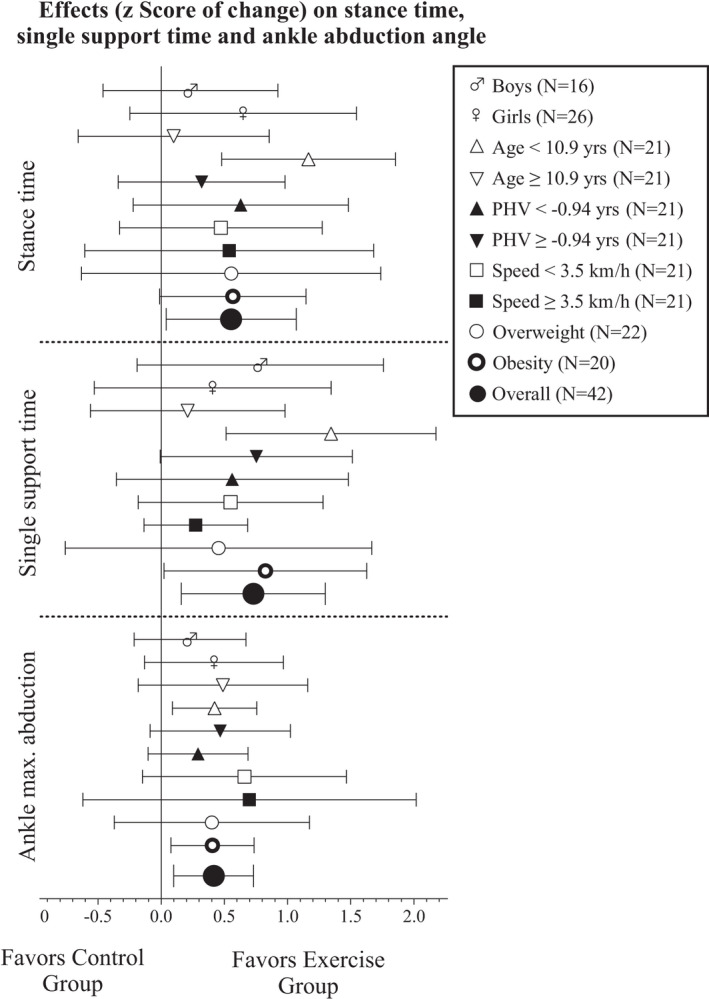
Per‐protocol overall and by groups (i.e., sex, age, peak high velocity [PHV], and gait speed) effects of the intervention on selected gait biomechanics variables. Dots represent *z* Score values of change with respect to the baseline mean and standard deviation. Each analysis was adjusted by baseline outcomes. Bars represent 95% confidence intervals. Age, PHV, and gait speed categories were calculated the median value. PHV calculated with the Moore's equations^21^ was used as indicator of maturational stage. Stance and single‐limb support time results were inverted (i.e., multiplied by −1) to facilitate the interpretation of the plot as favors exercise group

Sensitivity analyses are shown in Figure 2 and show how relevant confounder influence the effects of the exercise program on the outcomes that demonstrated significant between‐group differences (i.e., stance and single‐limb support times, and ankle abduction angle). For that purpose, we divided the sample in sub‐groups of sex, age, biological maturation, gait speed, and BMI categories.^27^, ^28^ Overall, the effects of the intervention were consistent across sex, maturation status, and gait speed, while a clear difference was observed between age groups and BMI categories. Significant changes in stance time, single‐limb support time and ankle abduction angle only occurred favor the EG in children over 11 years old and in the presence of obesity. Regarding musculoskeletal pain, there were no significant within‐group changes nor between‐group changes in pain prevalence or pain intensity (Appendix S1).

**FIGURE 3:**
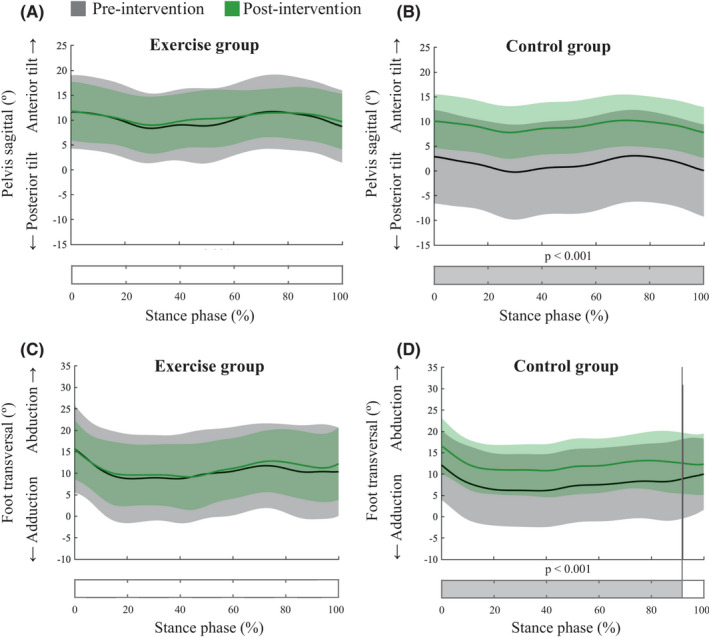
SPM1D analysis for the comparisons between pre‐ and post‐exercise in gait kinematic curves for each group (exercise and control groups). Solid lines represent mean and shaded areas standard deviation. Shaded area in the bars indicates significant differences between pre‐ and post‐exercise, which occurs when the SPM{t} values exceeded the alpha level threshold of 0.05

The SPM analysis showed an interaction effect between group and intervention effects for the sagittal angles of the pelvis and ankle transversal angles. The post hoc analysis showed no post‐exercise differences in the pelvis sagittal angle for the EG, while the CG significantly increased the anterior tilt angle during the entire stance phase (mean difference: 7.7º; cluster *p* < 0.001). There were no post‐exercise differences in the ankle abduction angle for the EG, while in the CG there was a significant increase from 0 to 92% of the stance phase (mean difference: 4.6º; cluster *p* < 0.001). All these results are shown in Figure 3.

The intention‐to‐treat analysis is shown in Appendix S1. Overall, effect sizes were attenuated, with the statistical significance in the spatiotemporal parameters disappearing and only remaining in the maximal ankle abduction angle (small effect size: −0.32 SDs; *p* = 0.026).

## 4 DISCUSSION

In the present study, we found that children with OW/OB who participated in our integrative neuromuscular training demonstrated no further deterioration of two biomechanical gait parameters that have previously been shown to be altered in this population,^2^ as well as deterioration being observed in our CG. Particularly, the EG experienced a lower increase in the stance and single‐limb support times compared to the CG. Furthermore, the EG maintained the same pelvic and ankle angles in the stance phase while walking, whereas the CG demonstrated an increase in pelvic anterior tilt and ankle abduction angles. All this suggest that the exercise program might be stopping the progression of some gait alterations, such as longer stance time, excessive pelvic anterior tilt and ankle abduction, in children with OW/OB.

Stance time and single‐support time experience a natural decrease from childhood to adulthood.^27^ However, neither our EG nor CG showed this natural phenomenon probably because of the OW/OB; moreover, the CG experienced even an increase in these two parameters. Compared with healthy normal‐weight children of the same age, our sample already presented a longer stance time at pre‐exercise (0.63 vs 0.67 s) confirming that an increase in this spatiotemporal parameter represents an alteration in the gait pattern.^29^ Walking with relatively longer steps while maintaining similar cadence is associated with lower mechanical efficiency, since it requires a higher force generation to re‐accelerate the center of mass in the step‐to‐step transition via a disruption in the normal stretch‐shortening cycle of muscles and tendons.^30^, ^31^ Based on all this evidence, findings from this study could be leading to positive effects of exercise on the mechanical efficiency of walking in children with OW/OB. However, future evidence including the energy cost and mechanical efficiency of walking should corroborate this hypothesis.

Our findings suggested some beneficial effects of our exercise program by stopping the progression of some gait kinematics alterations such as excessive pelvic anterior tilt and ankle abduction. Compared with healthy children with NW,^32^, ^33^ our participants presented excessive values in these two outcomes, which lead us to interpret the increase in the CG as a progression in the gait deterioration. An elevated pelvic anterior tilt together with an abducted position of the ankle are biomechanical alterations that usually occur simultaneously in what is known as the lower extremity movement impairment syndrome,^34^ and they are indicators of a hyperlordotic and pronated gait pattern.^32^, ^35^, ^36^ Lumbar hyperlordosis has been related to the presence of low back pain in childhood, and its progression through lifespan is considered a risk factor for severe spine pathologies such as herniated disk.^37^, ^38^ An excessive foot pronation is associated with overuse musculoskeletal disorders in adults such as knee pain and structural damage in the medial tibiofemoral cartilage.^39^, ^40^ Furthermore, to increase the ankle abduction in early‐ and mid‐stance phases of gait, as observed in the CG, it seems to increase the knee adduction moment, which is considered a major biomechanical factor for the development of knee osteoarthritis later in life.^41^, ^42^, ^43^ Despite the encouraging results of this study, it is important to note that the EG still demonstrated a worrying pelvic anterior tilt and ankle abduction angles during walking, so future studies should elucidate effective strategies to not only stop but also reverse these gait deteriorations. Moreover, biomechanical analyses of the pelvis present limitations in children with OW/OB, due to fat mass accumulation hampers the correct placement of skin‐mounted markers, and results should be considered with caution.

We only identified three previous studies testing the effects of exercise on gait biomechanics in children and adolescents with OW/OB.^8^, ^9^, ^10^ Unlike Delextrat et al,^10^ we could not test changes in walking speed since we maintained the speed that children had self‐selected pre‐exercise.^19^, ^28^ However, we found novel and promising results in the stance and single‐limb support times, which directly target gait biomechanical alterations typically experienced by this population.^2^ In terms of gait kinematics, both Horsak and Hainsworth's studies suggested positive effects of exercise in children and adolescents with OW/OB through improved lower‐limb alignment during the stance phase of walking.^8^, ^9^ In the present study, we did not find modifications in our EG toward a better alignment during gait, but a stabilization in the kinematics in comparison with the CG which increased their malalignments. A possible explanation for these contrasting findings is that participants from previous studies had already reached a mature gait, since they were over 13 years old on average, while our participants were still consolidating their gait pattern before puberty.^27^ In fact, we observed significantly greater effects in children near puberty (11–12 years old) than in children of 9 and 10 years old, although we acknowledge limitations in the statistical power. Based on these findings, we contemplate two potential explanations: (1) exercise interventions could be more effective in restoring an optimal gait biomechanics in young who have reached a mature gait, and (2) data in less mature gait presents too variability to find statistically significant results. Nevertheless, there is still little evidence available to draw firm conclusions and further research should confirm these observations.

Findings from this study are in line with those we reported in previous work with the same sample, which suggested positive functional changes in plantar pressure during walking induced by exercise.^13^ We found that the EG did not continue increasing the total plantar pressure surface, as the CG did, which is an indicator of flatfoot and pronated foot pattern during walking.^44^ Hyperpronation is normally linked with excessive foot abduction and in the current study, we found a stop in the progression of this abduction angle during the stance phase. We have three main hypotheses by which our exercise program could lead to improvements in gait biomechanics: (1) weight loss, (2) muscle strengthening, and (3) neuromuscular re‐education of movement patterns. Weight loss was already discarded in this previous study since these children did not reduced their body weight or BMI after the exercise program.^13^ Thus, the strengthening of key foot and ankle muscles (e.g., tibialis posterior and flexor hallucis longus) as well as a neuromuscular re‐education could be explaining these findings.^45^, ^46^ In fact, we found in a recent study that the present exercise program induced positive changes in the lower‐limb strength and functional movement quality of these children,^14^ which supports this hypothesis. Although further study is still needed on this topic, a considerable body of evidence begins to demonstrate that exercise interventions might be a potential treatment to stop and reverse the biomechanical alterations during walking in children and adolescents with OW/OB.^8^, ^9^, ^13^, ^47^, ^48^, ^49^

Exercise interventions might prevent the development of musculoskeletal disorders in children and adolescents with OW/OB. Some authors suggest two main pathomechanisms explaining the higher prevalence of musculoskeletal disorder with the presence of OW/OB^50^: (1) systemic inflammation due to adipose tissue accumulation and (2) biomechanical factors. A recent meta‐analysis has demonstrated that exercise reverses the inflammatory state normally observed in children and adolescents with OW/OB.^51^ Furthermore, findings from this research together with other studies demonstrate that exercise also is beneficial from a biomechanical perspective.^8^, ^13^, ^14^ Our results did not show any relevant change in lower‐limb musculoskeletal pain after the exercise program, something that could be expected since most of the children reported no pain or low/mild pain. However, future longitudinal studies should reveal whether the biomechanical modifications observed could prevent long‐term musculoskeletal diseases such as knee osteoarthritis in this population. It is important to mention that exercise is not the only available treatment, since weight loss programs by nutritional modifications and surgical interventions (e.g., subtalar arthroereisis and bariatric surgery) have also demonstrated positive effects on the gait biomechanics of children and adolescents with OW/OB.^52^, ^53^, ^54^ A possible intervention strategy could be to start with more conservative treatments, such as exercise and nutritional interventions, and prescribe surgical interventions only in the most extreme cases.

This study comes with a number of limitations. First, this study only reported gait spatiotemporal and kinematic outcomes, and additional biomechanical parameters such as gait kinetics, joint contact forces, or mechanical efficiency would provide a wider perspective of walking biomechanics in children with OW/OB. Second, the adherence to the exercise program was acceptable (i.e., 56% of children attending >70% of the prescribed 3 sessions per week) considering that children with OW/OB normally present low rates.^23^ However, further efforts are needed to increase the motivation of these children while practicing physical activity, in order to achieve long‐term adherence. Third, we used a standard skin‐mounted marker model in our 3‐D analysis of gait, which might introduce artifacts and inaccuracies due to the higher presence of fat mass in this population. Currently, there are more accurate approaches that personalize musculoskeletal models from radiographic images and should be considered in future studies of children with OW/OB.^55^, ^56^ Fourth, our marker model considered the foot as a rigid segment, which means that we could not gain insights into intersegmental foot motions in this population, such as midfoot eversion.^57^ Fifth, due to a non‐randomized assignment, the EG and CG presented baseline differences that might be influencing the results and reduce the external validity. However, all statistical analyses were adjusted for baseline values and sensitivity analyses were performed accounting for potential confounders (i.e., sex, age, maturational stage, and gait speed). Lastly, a post hoc statistical power analysis (G*Power tool) revealed that the minimum detectable effect size was 0.41 SDs to have less than 20% of chance to make a type II error with our sample size, and, thus, this study does not allow to detect small changes between groups.

## 5 PERSPECTIVE

This study shows that a 13‐week integrative neuromuscular training stopped the progression of some biomechanical alterations during walking in children with overweight/obesity. Findings of this research suggest that exercise leads to positive effects in the gait biomechanics of children and adolescents with OW/OB, which may ultimately contribute to the prevention of musculoskeletal disorders and the preservation of an optimal mechanical efficiency during walking in this population.

## CONFLICT OF INTEREST

Our results do not constitute endorsement by ACSM. We declare that the results of the study are presented clearly, honestly, and without fabrication, falsification, or inappropriate data manipulation.

## Supporting information

Appendix S1

## Data Availability

The data that support the findings of this study are available on request from the corresponding author. The data are not publicly available due to privacy or ethical restrictions
