# Supplementary material for: Effects of integrative neuromuscular training on the gait biomechanics of children with overweight and obesity
Source: Scand J Med Sci Sports. 2022 Apr 29;32(7):1119–30. doi: 10.1111/sms.14163 (PMC9540886; doi:10.1111/sms.14163)
Supplement: Supplementary file 1 — Appendix S1 [file SMS-32-1119-s001.docx]

| **Table S1.** Pre-intervention characteristics of the total sample and divided by intervention and control group for the intention-to-treat analysis | | | | |
| --- | --- | --- | --- | --- |
|  | All  (N = 50) | Exercise  (N = 25) | Control  (N = 25) | *P* |
|  | Mean ± SD | Mean ± SD | Mean ± SD |  |
| Age (years) | 10.77 ± 1.24 | 11.05 ± 1.18 | 10.50 ± 1.25 | 0.116 |
| Weight (kg) | 57.45 ± 12.28 | 61.94 ± 9.96 | 52.96 ± 12.91 | **0.008** |
| Height (cm) | 148.18 ± 8.60 | 150.25 ± 7.46 | 146.12 ± 9.29 | 0.090 |
| Body mass index (kg/m^2^) | 25.91 ± 3.57 | 27.33 ± 3.16 | 24.48 ± 3.43 | **0.004** |
| Gender N (%) |  |  |  | **0.009** |
| Girls | 31 (62) | 11 (44) | 20 (80) |  |
| Boys | 19 (38) | 14 (56) | 5 (20) |  |
| **Spatiotemporal parameters** |  |  |  |  |
| Cadence (steps/min) | 122.34 ± 11.46 | 120.37 ± 7.96 | 124.30 ± 14.03 | 0.230 |
| Stance time (cs) | 66.84 ± 5.41 | 67.45 ± 4.75 | 66.23 ± 6.03 | 0.433 |
| Single support time (cs) | 32.93 ± 2.50 | 33.22 ± 2.09 | 32.64 ± 2.87 | 0.419 |
| Double support time (cs) | 33.91 ± 3.81 | 34.23 ± 3.67 | 33.59 ± 3.99 | 0.561 |
| Step length (cm) | 50.74 ± 9.12 | 53.30 ± 8.66 | 48.17 ± 9.00 | **0.045** |
| Stride width (cm) | 13.84 ± 3.06 | 13.48 ± 2.64 | 14.19 ± 3.46 | 0.419 |
| **Kinematics: stance phase (º)** |  |  |  |  |
| Pelvis ROM sagittal | 4.63 ± 1.11 | 4.72 ± 1.19 | 4.54 ± 1.03 | 0.555 |
| Pelvis ROM transversal | 9.01 ± 3.60 | 10.02 ± 3.62 | 8.00 ± 3.34 | **0.046** |
| Knee ROM frontal | 6.05 ± 3.41 | 6.51 ± 4.22 | 5.59 ± 2.34 | 0.349 |
| Ankle max. plantarflexion | 60.32 ± 9.02 | 61.99 ± 8.81 | 58.65 ± 9.10 | 0.194 |
| **Kinematics: weight acceptance (º)** |  |  |  |  |
| Pelvis max. elevation | 3.45 ± 2.61 | 3.46 ± 2.79 | 3.44 ± 2.49 | 0.978 |
| Hip ROM frontal | 3.68 ± 2.12 | 4.01 ± 2.36 | 3.35 ± 1.83 | 0.276 |
| Knee ROM sagittal | 13.86 ± 5.21 | 14.54 ± 5.12 | 13.18 ± 5.32 | 0.364 |
| Ankle max. abduction | 13.97 ± 9.07 | 15.56 ± 9.40 | 12.38 ± 8.63 | 0.219 |
| SD = standard deviation; N = sample size;  Values are presented as mean ± SD or percentages. For continuous variables, p value was obtained by an independent samples T-test, whereas for categorical variables, p value was obtained by chi-square test.  Significant differences (p < 0.05) are highlighted in bold. | | | | |

| **Table S2.** Intention-to-treat intervention effects on gait biomechanics | | | | |
| --- | --- | --- | --- | --- |
| Adjusted post-intervention mean (95% CI) | | | | |
| Total sample = 50 | Intervention group  (N = 25) | Control group  (N = 25) | Groups difference (EG – CG) | *P* |
| **Spatiotemporal parameters** |  |  |  |  |
| Cadence (steps/min) |  |  |  |  |
| Raw score | 118.80 (115.45 to 122.15) | 114.89 (111.54 to 118.24) | 3.91 (-0.87 to 8.68) | 0.106 |
| z Score | -0.31 (-0.60 to -0.02) | -0.65 (-0.94 to -0.36) | 0.34 (-0.08 to 0.76) |  |
| Stance time (cs) |  |  |  |  |
| Raw score | 68.94 (67.13 to 70.75) | 71.36 (69.55 to 73.17) | -2.43 (-4.99 to 0.14) | 0.064 |
| z Score | 0.39 (0.05 to 0.72) | 0.84 (0.50 to 1.17) | -0.45 (-0.92 to 0.03) |  |
| Single support time (cs) |  |  |  |  |
| Raw score | 34.35 (33.43 to 35.28) | 35.62 (34.69 to 34.69) | -1.26 (-2.57 to 0.05) | 0.058 |
| z Score | 0.57 (0.20 to 0.94) | 1.07 (0.70 to 1.44) | -0.5 (-1.03 to 0.02) |  |
| Double support time (cs) |  |  |  |  |
| Raw score | 34.64 (33.59 to 35.7) | 35.69 (34.63 to 36.74) | -1.04 (-2.53 to 0.45) | 0.166 |
| z Score | 0.19 (-0.08 to 0.47) | 0.47 (0.19 to 0.74) | -0.27 (-0.67 to 0.12) |  |
| Step length (cm) |  |  |  |  |
| Raw score | 53.47 (51.6 to 55.34) | 53.36 (51.49 to 55.23) | 0.11 (-2.59 to 2.81) | 0.937 |
| z Score | 0.30 (0.09 to 0.50) | 0.29 (0.08 to 0.49) | 0.01 (-0.28 to 0.31) |  |
| Stride width (cm) |  |  |  |  |
| Raw score | 14.20 (13.32 to 15.09) | 13.30 (12.42 to 14.18) | 0.90 (-0.35 to 2.16) | 0.154 |
| z Score | 0.12 (-0.17 to 0.41) | -0.18 (-0.47 to 0.11) | .3 (-0.12 to 0.71) |  |
| **Kinematics: stance phase** |  |  |  |  |
| Pelvis ROM sagittal (º) |  |  |  |  |
| Raw score | 4.24 (3.75 to 4.73) | 3.95 (3.46 to 4.45) | 0.29 (-0.41 to 0.99) | 0.414 |
| z Score | -0.35 (-0.80 to 0.09) | -0.61 (-1.06 to -0.17) | 0.26 (-0.37 to 0.89) |  |
| Pelvis ROM transversal (º) |  |  |  |  |
| Raw score | 9.38 (7.62 to 11.13) | 8.11 (6.35 to 9.86) | 1.27 (-1.26 to 3.80) | 0.318 |
| z Score | 0.10 (-0.39 to 0.59) | -0.25 (-0.74 to 0.24) | 0.35 (-0.35 to 1.06) |  |
| Knee ROM frontal (º) |  |  |  |  |
| Raw score | 8.11 (6.89 to 9.33) | 7.69 (6.47 to 8.91) | 0.42 (-1.31 to 2.16) | 0.624 |
| z Score | 0.61 (0.25 to 0.96) | 0.48 (0.12 to 0.84) | 0.12 (-0.38 to 0.63) |  |
| Ankle max. plantarflexion (º) |  |  |  |  |
| Raw score | 55.91 (53.79 to 58.03) | 57.27 (55.15 to 59.39) | 1.36 (-4.39 to 1.66) | 0.370 |
| z Score | 0.49 (0.25 to 0.72) | 0.34 (0.10 to 0.57) | 0.15 (-0.18 to 0.49) |  |
| **Kinematics: weight acceptance** |  |  |  |  |
| Pelvis max. elevation (º) |  |  |  |  |
| Raw score | 2.27 (1.48 to 3.06) | 1.82 (1.03 to 2.61) | 0.46 (-0.66 to 1.57) | 0.417 |
| z Score | -0.45 (-0.75 to -0.15) | -0.62 (-0.93 to -0.32) | 0.17 (-0.25 to 0.60) |  |
| Hip ROM frontal plane (º) |  |  |  |  |
| Raw score | 3.77 (3.08 to 4.45) | 3.29 (2.60 to 3.97) | 0.48 (-0.50 to 1.46) | 0.327 |
| z Score | 0.04 (-0.28 to 0.37) | -0.18 (-0.51 to 0.14) | 0.23 (-0.23 to 0.69) |  |
| Knee ROM sagittal (º) |  |  |  |  |
| Raw score | 13.77 (12.41 to 15.14) | 14.42 (13.05 to 15.78) | -0.64 (-2.58 to 1.29) | 0.507 |
| z Score | -0.02 (-0.28 to 0.25) | 0.11 (-0.16 to 0.37) | -0.12 (-0.49 to 0.25) |  |
| Ankle max. abduction (º) |  |  |  |  |
| Raw score | 15.48 (13.70 to 17.25) | 18.37 (16.60 to 20.14) | -2.89 (-5.42 to -0.37) | **0.026** |
| z Score | 0.17 (-0.03 to 0.36) | 0.49 (0.29 to 0.68) | -0.32 (-0.60 to -0.04) |  |
| CI = confidence interval; n = sample size; EG = exercise group; CG = control group; cs= centiseconds.  A one-way analysis of covariance (ANCOVA) was used to test raw and z-score differences between the EG and CG at post-exercise, adjusting for pre-exercise values. Z-score values in the “group difference” column indicates how many standard deviations has changed the EG compared to the CG, and can be interpreted as an effect size indicator: 0.2 – 0.5 SDs = small effect size; 0.5 – 0.8 SDs = medium effect size; and ≥ 0.8 = large effect size (e.g., 0.51 z-score means that the EG has changed +0.51 standard deviations compared to the CG, which is a medium effect size). Z-score values in both “exercise group” and “control group” columns indicates how many standard deviations has changed each group with respect to itself between the pre- and post-exercise assessment (e.g., 0.51 z-score in the “exercise group” column means that the EG is 0.51 standard deviations higher at post-exercise than at pre-exercise). Adjusted means and confidence intervals of the mean are represented. Significant differences (p < 0.05) are highlighted in bold. | | | | |

**Figure S1.** Intervention effects on musculoskeletal pain.

**Three-dimensional gait biomechanics: data processing and calculation.** First, an upright static trial was used to create the lower limbs segment (i.e., pelvis, femur, shank and foot) and the joint centers (i.e., pelvis, hip, knee and ankle) posteriorly used in the motion trials. Second, marker trajectories were filtered using a low-pass Butterworth filter with a cut-off frequency of 7 Hz. We selected this filter frequency after performing a residual analysis with different cut-off frequencies (i.e., 3 to 10 Hz) and considering previous literature in this population (1, 2). Third, ﻿joint angular displacements of pelvis, hip, knee and ankle in all three planes (sagittal, frontal and transversal) were calculated as the relative orientation of the distal segment to the proximal segment. After verify that there were no overall kinematic asymmetries between both lower limbs, we decided to analyze the right lower limbs for all participants. Fourth, gait events (heel contact and toe off) were automatically calculated in Visual 3D based on the kinematic data using previously described algorithm in gait analysis (3). Fifth, based on these events the gait cycle was divided in stance phase (from right heel contact to right toe off) and weight acceptance phase (from right heel contact to left toe off), and subsequently spatiotemporal parameters were calculated considering the gait speed and height of participants (4). Sixth, range of motion (ROM) and maximum displacement angles were calculated in key joints (i.e., pelvis, hip, knee and ankle), planes and gait phases previously reported in the literature of this population (5, 6). Seventh, we detected the most representative stride out of the seven strides we captured in each participant using the approach of ﻿Sangeux and Polak (7). From this representative stride we selected the above-mentioned spatiotemporal parameters, ROM, maximum displacement and kinematics curves normalized to 100% of the stance phase. We focused on the stance phase because a recent systematic review reported gait biomechanical alterations in this phase but not in swing phase for children with OW/OB (5).

**REFERENCES**

1. Shultz SP, D’Hondt E, Fink PW, Lenoir M, Hills AP. The effects of pediatric obesity on dynamic joint malalignment during gait. *Clin Biomech*. 2014;29(7):835–8.

2. Lerner ZF, Browning RC. Compressive and shear hip joint contact forces are affected by pediatric obesity during walking. *J Biomech*. 2016;49(9):1547–53.

3. Zeni JA, Richards JG, Higginson JS. Two simple methods for determining gait events during treadmill and overground walking using kinematic data. *Gait Posture*. 2008;27(4):710–4.

4. Lythgo N, Wilson C, Galea M. Basic gait and symmetry measures for primary school-aged children and young adults. II: Walking at slow, free and fast speed. *Gait Posture*. 2011;33(1):29–35.

5. Molina‐Garcia P, Migueles JH, Cadenas‐Sanchez C, et al. A systematic review on biomechanical characteristics of walking in children and adolescents with overweight/obesity: Possible implications for the development of musculoskeletal disorders. *Obes Rev*. 2019;20(7):1033–44.

6. Horsak B, Schwab C, Baca A, et al. Effects of a lower extremity exercise program on gait biomechanics and clinical outcomes in children and adolescents with obesity: A randomized controlled trial. *Gait Posture*. 2019;70:122–9.

7. Sangeux M, Polak J. A simple method to choose the most representative stride and detect outliers. *Gait Posture*. 2015;41(2):726–30.
